# Mental Derangement

**Published:** 1854-11

**Authors:** Roger G. Perkins

**Affiliations:** Senior Assistant Physician, New York Lunatic Asylum, Blackwell’s Island


### DEPARTMENT OF SELECTIONS.

### MENTAL DERANGEMENT—ITS SYMPTOMS AND TREATMENT.

BY ROGER G. PERKINS, M. D.

Senior Assistant Physician, New York Lunatic Asylum, Blackwell's Island.

Insanity is a term applied, by those who use it correctly, to that state of mind in which healthy action is suspended. This is, perhaps, as complete a definition as can be given of the disease. It has been defined "a loss of mental freedom," "a destruction of spontaniety in psychological action." These definitions, both meaning the same thing, are neither of them admissible, as they embrace sleep-waking and dreaming.

Locke's definition was, that madness consisted in "reasoning from false premises." Cullen calls insanity a false perception and consequent erroneous judgment. Prichard has well remarked, that both these definitions embrace but one form of mental disease, viz. hallucination. His own definition is as follows: "A chronic disease, manifested by deviations from the healthy and natural state of the mind; such deviations existing either in moral perversion, or a disorder of the feelings, affections, and habits of the individual, or, in intellectual disarrangement; which last is sometimes partial, as in monomania, affecting the understanding only in particular trains of thought; or general, and accompanied by excitement, namely, in mania or raving madness; or lastly, confounding or destroying the connections or associations of ideas, and producing as tate of incoherence." The main objection to this definition is the use of the word *chronic*; insanity is oftentimes acute, both as to time and phenomena. Again, the definition is too long; and, long as it is, does not include the delirium of fever, the coma of apoplexy, or the lethargy sometimes following epilepsy, all of which diseased conditions belong to a mind not "*sana in corpore sano*," and, consequently, are improperly considered as anything else than forms of insanity. The great difficulty found by many writers on this subject, in attempting to define this disease, has been lest they should include the delirium of fever; but, without doubt, any one of these authors, if called upon to testify in the case of a man who during the delirium of fever had murdered his child, would urge his acquittal on the ground of temporary insanity. Throughout the whole extent of the writer's reading, there has been found no definition which seemed so proper as that which stands at the head of this article, and which naturally suggested itself to him, as it without doubt has to many others, at the very outset of his undertaking.

All forms of mental derangement can be included for description in four classes: 1st. Mania. 2d. Melancholia. 3d. Dementia. 4th. Moral Insanity.

It is proposed to consider these four forms of mental disease separately.

I. Mania is divided into general and partial, each of which divisions contains several varieties.

The word is derived from the Greek verb signifying to rave. It has been in use as applied to this form of disease from the earliest periods of the history of medicine. Its appropriateness has never been doubted.

A patient upon whom general mania is making its approach, generally exhibits nearly the following symptoms immediately before the full development of the disease: Every natural thought and feeling is exaggerated. At first, this is scarcely noticeable; but, after a short time, the exaltation becomes more and more manifest; soon we observe former objects of quiet and unobtrusive affection are treated with the most persuasive and endearing fondness, and at the same time that those who may once have incurred the displeasure of the patient, are spoken of with a bitterness and angry vindictiveness totally inconsistent with the previous character of the individual. The patient is evidently becoming more and more a creature of impulse, and cannot fix his attention upon the ordinary employments of the day, but is wandering with strides increasing in rapidity from one thing to another. As the excitement increases, the wildness and often redness of the eye begin to attract attention. The heat of head is sometimes found to be considerable, though the patient has, perhaps, made no complaint of pain; the pulse may be full and frequent, or small and quick; the tongue is sometimes furred, the bowels costive, the skin nearly normal; not unfrequently the hands and feet, perhaps arms and legs, are cold and clammy; the muscles of the face are beginning to seem more prominent, and it may be they act spasmodically; the expression of the countenance is rapidly beginning to change; and the language, growing more and more rapid, loud, and boisterous, is interrupted by strong exertions to procure self-control, and is, perhaps, not unmixed with loud laughing or tears, as the subjects of conversation be gay or sad. At this time a wondering question, or provoked remonstrance, falls upon the patient's ear from some one who witnesses his strange actions. The patient flies at once into a passion; his abuse is loud and terrible; horrible blasphemy is poured forth with extreme rapidity; and, wildly gesticulating over the extended arms of those who seek to curb his fury, he heaps the curses of all the gods and fiends upon the devoted object of his anger. The ideas follow so quickly upon one another, he cannot finish the expression of one before another claims his utterance, and the first is left unfinished; at last, so fast do the ideas throng through his mind, he can no longer be coherent; in his eagerness to express them all, confusion follows, and the patient is a raving maniac. This is acute mania.

It is generally three or four days before the symptoms above recited render it necessary to confine the patient, and during this time much may be done to lessen the violence of the expected attack; but of this, further on. The occurrence of acute mania, as above described, is preceded by some months, perhaps only weeks, of ill-health, slight depression of spirits, headache, sleeplessness, and anxiety, all of which precursory symptoms are vividly remembered by the friends of the patient, after it is, perhaps, too late for their treatment. Where the nervous temperament predominates, and particularly in those in which there is hereditary taint of insanity, the physician can hardly be too careful in his attentions to patients complaining of headache, sleeplessness, etc., as, by proper means, insanity is many times averted. After the patient has become raving in his madness; when the rapid incoherence, with wild gesticulations and other symptoms, have become perfect masters of the patient, and total loss of self-control has ensued, the condition of the unfortunate maniac is pitiful indeed. With few exceptions, the propensity to rend has full force; the patient speedily disrobes himself, and his clothes are in tatters in an hour. Windows (if within his reach) are shivered; and, reckless of the wounds upon his hands, caused by the broken glass, the doors and walls resound to his impetuous beating, while the air is filled with howling blasphemy.—The natural evacuations are strewed upon the floor and smeared over the walls; articles of furniture are broken; mattrasses emptied of their contents; bed-clothing torn to ribands; when, finally, finding more within his reach, the patient throws himself upon the rubbish he has formed, and, amid tears and laughter, buries himself beneath it. Upon the entrance of another, he may be calm for a moment; as soon as he opens his mouth to speak, however, there rushes from it a storm of curses and reproaches. It is not possible to say how long this state of things would continue, if uninterrupted by medication, as the deep injustice of an experiment upon such a case has not been attempted: it would probably last until the patient should sleep through exhaustion, or become weak through abstinence. From *fitful* sleep he wakes but to continue his ravings, and from the food demanded he gains additional strength to do so. Long before this, however, proper medicine has been used, and its effects begin to manifest themselves. The hallucinations are generally those of fear and suspicion. The suspicion may fix itself upon the nearest friend, in fact, it generally does so, and the feeling may become so strong as to reach an homicidal point.

The fear, in almost all cases, is for his own life, and under these circumstances the patient almost always attempts suicide.

The state of bodily fear in which he is, is too intense; and rather than endure it any longer, he attempts to place himself beyond the seach of it by self-destruction.

Chronic general mania pursues the same course as the acute, but not with so great violence, and with occasional complete remissions. During these lucid moments, and short moments they often are, the patient not unfrequently acknowledges his illness, is thankful for kindness, and sometimes profuse in his apologies and expressions of obligation.

As will be easily conceived, this continued raving cannot long exist without, sooner or later—as the constitution of the individual will admit—the supervention of extreme emaciation and pallor. The earnest, fixed, and glassy look soon takes full possession of the eyes; objectless and almost constant movements of the hands, often accompanied with the twitching of the muscles of the face, are now present. The appetite, which while the mania was acute was but little, almost always entirely gone, oftentimes becomes voracious. The bowels, before obstinate in constipation or diarrhœa, are now, perhaps, regular. The skin assumes an unnatural dryness, hardness, and color. The pulse is ranging from 90 to 100, full and strong—or from 108 to 116, small weak, and irritable. The tongue, no longer much furred, is nearly normal—though, in cases of small and irritable pulses, the papillæ are elongated. The temperature of the body of the patient is generally nearly normal. The pupils largely dilated.

To be continued.

